# Targeting nerve growth factor, a new option for treatment of osteoarthritis: a network meta-analysis of comparative efficacy and safety with traditional drugs

**DOI:** 10.18632/aging.202232

**Published:** 2020-12-03

**Authors:** Ziqin Cao, Jian Zhou, Zeling Long, Yihan Li, Jingjing Sun, Yingquan Luo, Wanchun Wang

**Affiliations:** 1 Department of Orthopedics, The Second Xiangya Hospital, Central South University, Changsha, Hunan, China; 2 Department of Orthopedics, Mayo Clinic, Rochester, MN 55905, USA; 3 Department of Orthopedics, University of California, Davis, CA 95817, USA; 4 Department of Anesthesiology, Second Affiliated Hospital, School of Medicine, Zhejiang University, Hangzhou, Zhejiang, China; 5 Department of General Medicine, The Second Xiangya Hospital, Central South University, Changsha, Hunan, China

**Keywords:** osteoarthritis, nerve growth factor, NSAIDs, opioids, pain relief

## Abstract

Objective: Osteoarthritis (OA) is the most common joint disease and leading cause of pain and disability in the elderly population. Most guidelines recommend the use of non-steroidal anti-inflammatory drugs (NSAIDs) and opioids for the non-operative treatment of OA. Monoclonal nerve growth factor (NGF) antibodies are new drugs with the potential to provide pain relief and functional improvement in OA. We compared the efficacy (pain reduction and functional improvement), and safety of monoclonal NGF antibodies with NSAIDs and opioids in the treatment of OA with a Bayesian network meta-analysis.

Results: 38 articles, comprising 41 trials and 20489 patients with OA were included. Overall from the network meta-analysis, anti-NGFs were the most effective drugs for pain relief (Standardized Mean Difference or SMD compared with placebo 4.25, 95% CI 2.87 to 5.63, Surface Under the Cumulative RAnking curve or SUCRA=93.7%) and for functional improvement (SMD 4.90, 95% CI 3.46 to 6.33, SUCRA=98.3%). Although anti-NGFs were associated with higher risk of peripheral sensation abnormality (paresthesia and pruritus), they were not associated with higher risk of other AEs (headaches and nausea) or with higher withdrawal rates related to AEs.

Conclusions: Monoclonal NGF antibodies provide significantly greater pain relief and functional improvement in OA compared to NSAIDs and opioids. Monoclonal NGF antibodies are not associated with severe AEs. More studies are needed to confirm these findings.

Methods: PubMed, CNKI, Web of Science, Scopus, Embase and Cochrane Library databases were searched for relevant studies (OA treated with anti-NGFs, opioids, selective COX-2 inhibitors or NSAIDs) published between January 1999 to January 2020. Bayesian network and conventional meta-analyses were conducted. Pain relief, functional improvement and AEs were assessed.

## INTRODUCTION

Osteoarthritis (OA) is the most common joint disease. It is the leading cause of pain and disability in the elderly population. It is estimated that at least 300 million people worldwide suffer from OA [1]. OA is a chronic disease characterized by cartilage degeneration, osteophyte formation, and synovial inflammation. The most common joints affected are the knee, hip, and hand. The pain and subsequent physical dysfunction caused by OA are associated with increased mortality risk [2]. In addition, because of the high prevalence of the disease, treatment presents an economic burden to society [3]. To treat the pain and other symptoms, most guidelines recommend the use of non-steroidal anti-inflammatory drugs (NSAIDs) and opioids [1]. However, the use of these drugs is limited by tolerability and safety concerns [4].

In the 1950s Levi-Montalcini et al. [5] discovered nerve growth factor (NGF), which was the first molecule in the class now known as the neurotrophins. Subsequent studies confirmed the important role of NGF in the development of sensory neurons responsible for nociception and temperature sensation. Studies showed that the withdrawal or inhibition of NGF decreases the sensitivity of peripheral nociceptors and down-regulates expression of neuropeptide transmitters [6]. Clinically this can result in significant pain relief. Based on these observations, numerous monoclonal NGF antibodies have been developed as potential alternative analgesics to NSAIDs and opioids in conditions with chronic severe pain. Three monoclonal NGF antibodies have been tested in clinical trials in OA, tanezumab, fulranumab and fasinumab. All trials have shown substantial and significant efficacy [7–15].

Numerous systematic reviews and meta-analyses have been conducted to investigate the efficacy and safety of NSAIDs and/or opioids for treatment of OA pain. The goal of our current network meta-analysis was to include the NGF antibodies in this comparison. Based on a recent network meta-analysis that showed NSAIDs and opioids are efficacious in pain relief in OA, we included 13 drugs in our network meta-analysis. These drugs were divided into 5 groups based on activity and mechanism of action: anti-NGFs (tanezumab, fulranumab, fasinumab), potent opioids (oxycodone, hydromorphone, oxymorphone), weak opioids (tramadol), selective COX-2 inhibitors (celecoxib, etoricoxib, rofecoxib), and traditional NSAIDs (ibuprofen, naproxen, diclofenac, paracetamol/acetaminophen). In a Bayesian network meta-analysis of 41 trials in OA, we assessed drug efficacy, including pain reduction and physical function improvement, and safety.

## RESULTS

### Study selection

**Figure 1:**
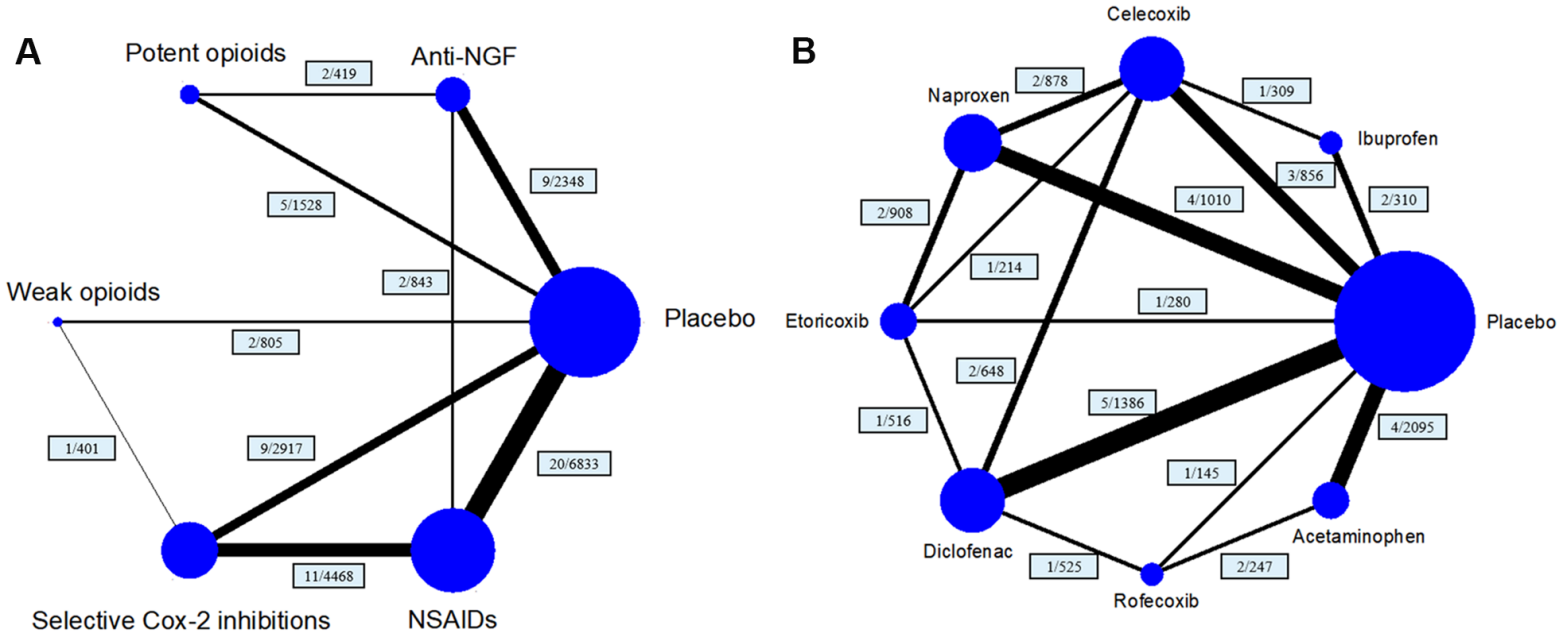
**Structure of network formed by interventions.** The lines between treatment nodes indicate the direct comparisons made within randomised controlled trials. Numbers (n/n) near the line indicate ‘number of trials/number of participants’ of the related comparisons. (**A**) the network plot of main network metanalysis. (**B**) the network plot of subgroup analysis comparing different selective COX-2 inhibitor and traditional NSAIDs.

This network meta-analysis was conducted based on the Preferred Reporting Items for Systematic Reviews and Meta-Analyses (PRISMA) guidelines [16]. A total of 38 articles covering 41 trials [17–45], were included. The selection criteria are shown in Supplementary Figure 1. Five treatment arms (anti-NGFs, potent opioids, weak opioids, selective COX-2 inhibitors, and NSAIDs) were included in the network of the main analysis, and eight treatment arms (celecoxib, etoricoxib, rofecoxib, ibuprofen, naproxen, diclofenac, paracetamol/acetaminophen and placebo) were included in the network of the subgroup analysis (Figure 1).

### Study characteristics

A total of 20489 patients were included in the studies. Among the 38 eligible articles, only one study with 385 patients was on hand-joint OA. Across all the trials, the mean age of patients was 61.25 years (range 57.41 to 70.00 years), the percentage of male patients was 32.26% (range 19.57% to 54.03%), and the median follow-up was 84 days (Interquartile range or IQR 42–84 days). The numbers of assessed patients for each treatment were NSAIDs 5408, selective COX-2 inhibitors 4131, anti-NGFs 3108, weak opioids 1405, and potent opioids 1274.

The baseline characteristics of the included studies are shown in Supplementary Table 1. The methodological quality and risk of bias were evaluated for all included trials (Supplementary Table 2). Based on these results, the main contributing factors to the risk of bias were performance bias, selection bias, and attrition bias. A funnel plot was used to assess evaluate effect of small sample size. The funnel plot was presented in Supplementary Figure 3. The detailed results of inconsistency of network comparison were presented in Supplementary Figures 4, 5.

### Primary efficacy endpoint

### Direct pair-wise meta-analysis

All the drugs except the potent opioids were superior to placebo for pain relief (see Table 1 for pairwise meta-analyses vs placebo). Notably, anti-NGFs showed a significant effect for pain relief (SMD 4.817, 95% Confidence Interval or CI 3.077 to 6.557).

**Table 1:** Characteristics of the included comparisons and the results of direct pair-wise meta-analysis (No. of patients, number of patients included; No. of trials, number of trials included into direct pair-wise meta-analysis; SMD, standardised mean difference).

**Comparison (compared with placebo)**	**No. of trials**	**No. of patients**	**Target joint**	**Mean age (Range)**	**Male,%**	**Heterogeneity for pain relief, I^2^**	**SMD (95%CI) for pain relief**	**Heterogeneity for function improvement, I^2^**	**SMD (95%CI) for function improvement**
Anti-NGF	9	2348	Hip and Knee	59.98 (57.41-62.32)	36.67	99.30%	4.182 (3.778,4.586)	99.30%	5.108 (3.165,7.051)
Potent opioids	5	1528	Hip and Knee	61.1 (57.41-65.52)	36.62	99.60%	0.807 (-1.527,3.140)	99.50%	1.058 (-1.012,3.127)
Weak opioids	2	805	Hip and Knee	59.09 (58.10-60.02)	37.23	98.3%%	3.451 (1.722,5.180)	98.50%	3.181 (1.466,4.896)
Selective cox-2 inhibition	9	2917	Hip and Knee	62.81 (60.02-64.77)	28.63	92.20%	4.775 (2.836,6.714)	92.20%	4.528 (2.642,6.415)
NSAIDs	20	6833	Hand, Hip and Knee	62.55 (58.66-70.00)	29.90	99.60%	4.775 2.573(1.789,3.357)	99.50%	2.677 (1.870,3.484)

### Network meta-analysis

A total of 38 trials were analyzed. No significant inconsistency was found in loop-inconsistency estimates, node-split tests, and global inconsistency tests. The consistency model was statistically significant compared with the inconsistency model.

**Figure 2:**
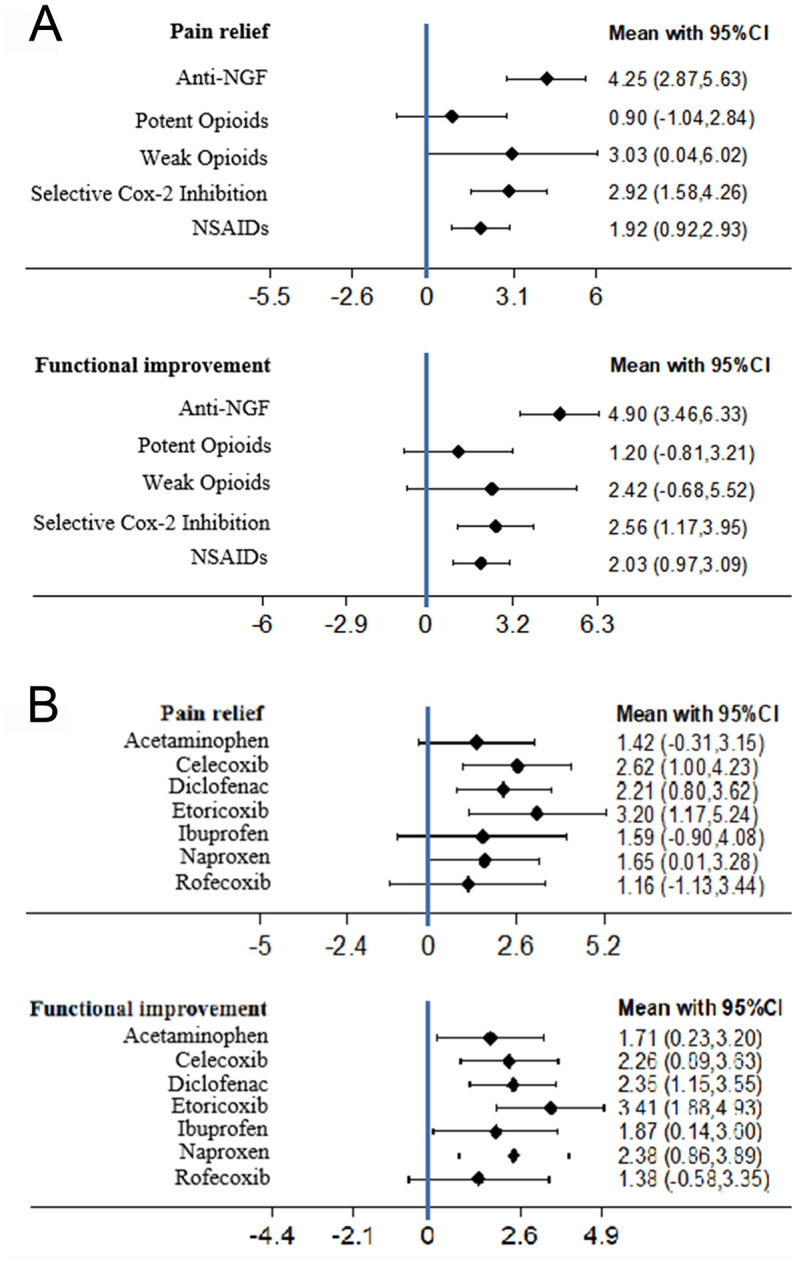
(**A**) The forest plots of pain relief and function improvement for main network meta-analysis. (**B**) The forest plots of pain relief and function improvement for subgroup analysis comparing different selective COX-2 inhibitor and traditional NSAIDs (SMD, standardised mean difference).

Anti-NGFs were the most efficacious drugs for pain relief (SMD compared with placebo 4.25, 95% CI 2.87 to 5.63). The potent opioids had the lowest efficacy and no significant effect (SMD 0.90, 95% CI -1.04 to 2.84) (Figure 2 and Table 2). Based on the SUCRA value, anti-NGFs were the most efficacious drugs for pain relief (SUCRA=93.7%), followed by selective COX-2 inhibitors (SUCRA=69.0%), and lastly opioids (SUCRA=67.3%). Anti-NGF were not significantly different than selective COX-2 inhibitor (SMD 1.33, 95% CI [-0.55 to 3.21]) and weak opioid drug (SMD 1.22, 95% CI [-2.07 to 4.51]), while it demonstrated better pain relief than NSAIDs (SMD 2.33, 95% CI [0.69 to 3.96]) and potent opioid drug (SMD 3.35, 95% CI [1.16 to 5.53]). The details of the SURCA rank are shown in Supplementary Table 3.

**Table 2:** Detailed results of network meta-analysis for pain (Red) and function (Blue) (Data are standardised mean difference, from the top left to the bottom right, higher comparator vs lower comparator, and their related 95% CI).

Anti-NGF	2.34 (0.38,4.29)	2.48 (-0.93,5.89)	2.87 (1.16,4.57)	3.70 (1.43,5.96)	4.90 (3.46,6.33)
1.33 (-0.55,3.21)	Selective cox-2 inhibition	0.15 (-3.09,3.38)	0.53 (-0.77,1.84)	1.36 (-1.07,3.80)	2.56 (1.17,3.95)
1.22 (-2.07,4.51)	-0.11 (-3.23,3.00)	Weak opioids	0.39 (-2.83,3.60)	1.22 (-2.47,4.91)	2.42 (-0.68,5.52)
2.33 (0.69,3.96)	1.00 (-0.24,2.23)	1.11 (-1.99,4.20)	NSAIDS	0.83 (-1.42,3.09)	2.03 (0.97,3.09)
3.35 (1.16,5.53)	2.02 (-0.33,4.36)	2.13 (-1.44,5.69)	1.02 (-1.15,3.19)	Potent opioids	1.20 (-0.81,3.21)
4.25 (2.87,5.63)	2.92 (1.58,4.26)	3.03 (0.04,6.02)	1.92 (0.92,2.93)	0.90 (-1.04,2.84)	Placebo

Subgroup analysis did not reveal any substantial change after excluding the 10 trials that were not commercially funded. Anti-NGFs were still the most efficacious drugs for pain relief (SUCRA 92.4%, SMD=4.30, 95% CI 2.85 to 5.74) (Supplementary Table 5).

### Secondary efficacy endpoint

### Direct pair-wise meta-analysis

The anti-NGFs and selective COX-2 inhibitors significantly improved physical function compared to placebo while the potent opioids had no significant effect (see Table 1 for pairwise meta-analyses vs placebo). Anti-NGFs had the highest efficacy for functional improvement (SMD 5.108, 95% CI 3.165 to 7.051).

### Network meta-analysis

A total of 38 trials were analyzed. No significant inconsistency was reported, and the consistency model was statistically significant compared to the inconsistency model. Anti-NGFs had the highest efficacy for functional improvement (SMD 4.90, 95% CI 3.46 to 6.33) (Figure 2). The potent opioids had the lowest efficacy and no significant effect (SMD 1.20, 95% CI -0.81 to 3.21) (Figure 2 and Table 2). Based on SUCRA, the anti-NGFs were the most efficacious drugs for functional improvement (SUCRA=98.3%), followed by selective COX-2 inhibitors (SUCRA=63.5%) and opioids (SUCRA=56.7%) (Supplementary Table 3). In the subgroup analysis there was no substantial change after excluding the 10 trials that were not commercially funded. Anti-NGFs still had the highest efficacy for functional improvement (SUCRA 97.6%, SMD 4.96, 95% CI 3.42 to 6.50). Apart from the selective COX-2 inhibitor group (SMD 2.34, 95%CI [-0.38 to 4.29]) and weak opioid group (SMD 2.48, 95%CI [-0.93 to, 5.89]) which were not significantly different than Anti-NGF drug, other groups all demonstrated less function improvement (Supplementary Table 3). There was no significant difference in the subgroup analysis of the trials with commercial funding. Anti-NGF still had the highest efficacy (SUCRA 97.6%, SMD 4.96, 95% CI [3.42 to 6.50]) (Supplementary Table 5).

### Primary safety endpoint

### Direct pair-wise meta-analysis

There were significant increases in withdrawal rates related to AEs with anti-NGFs and opioids compared to placebo, but not with selective COX-2 inhibitors (Odds Ratio or OR 0.742, 95% CI: 0.436 to 1.261) (Table 3).

**Table 3:** Adverse effects of different treatment compared with placebo according to direct pair-wise meta analysis and network meta-analysis (AE, adverse effect; PSA, peripheral sensation abnormality; SUCRA, surface under the cumulative ranking.).

**Treatment**		**Heterogeneity for direct comparison, I2(%)**	**OR(95%CI)**		**SURCA(%)**
			**Direct comparison**	**Network comparison**	
Withdrawal due to AEs					
	Placebo	Reference	Reference	Reference	82.3
	Anti-NGF	33.1	1.677(1.045,2.692)	1.36 (0.82,2.27)	53.5
	Potent Opioids	36.8	5.265(3.705,7.482)	8.63 (5.42,13.77)	0.1
	Weak Opioids	68.4	2.798(1.348,5.807)	3.27(1.89,5.66)	20.1
	Selective cox-2 inhibition	56.9	0.742(0.436,1.261)	0.89 (0.64,1.24)	93.5
	NSAIDs	29.6	1.272(1.028,1.573)	1.36(1.04,1.77)	50.5
Headache AEs					
	Placebo	Reference	Reference	Reference	53.3
	Anti-NGF	0.0	0.970(0.634,1.483)	1.03 (0.68,1.57)	49.3
	Potent Opioids	0.0	1.283(0.897,1.837)	1.19 (0.78,1.82)	29.2
					
	Weak Opioids	49.8	1.305(0.709,2.399)	1.41 (0.88,2.25)	12.4
	Selective cox-2 inhibition	0.0	0.748(0.528,1.060)	0.93 (0.68,1.27)	65.4
	NSAIDs	1.3	0.928(0.733,1.175)	0.82 (0.63,1.06)	90.3
Nausea AEs					
	Placebo	Reference	Reference	Reference	74.2
	Anti-NGF	33.1	0.962(0.504,1.837)	0.79 (0.41,1.53)	88.7
	Potent Opioids	36.8	4.519(3.212,6.358)	6.33 (3.37,11.90)	3.3
	Weak Opioids	68.4	3.131(2.054,4.775)	3.90 (1.82,8.36)	17.0
	Selective cox-2 inhibition	56.9	0.825(0.398,1.708)	0.99 (0.64,1.52)	75.8
	NSAIDs	29.6	1.432(0.947,2.165)	1.48 (1.04,2.13)	41.1
PSA AEs					
	Placebo	0.0	Reference	Reference	82.7
	Anti-NGF	0.0	4.184(2.010,8.707)	3.64 (1.87,7.10)	30.7
	Potent Opioids	0.0	5.331(2.731,10.407)	5.39 (2.41,12.06)	14.2
	Weak Opioids	0.0	8.371(2.935,23.870)	5.25 (1.95,14.15)	15.4
	Selective cox-2 inhibition	0.0	0.777(0.021,28.890)	0.96 (0.39,2.39)	82.6
	NSAIDs	82.8	0.966(0.633,1.473)	1.13 (0.67,1.90)	74.4

### Network meta-analysis

In the withdrawal related to AEs network, a total of 36 trials were assessed. No significant inconsistency was reported. Significantly higher withdrawal rates related to AEs were reported with potent opioids (OR 8.63, 95% CI 5.42 to 13.77), weak opioids (OR 3.27, 95% CI 1.89 to 5.66) and NSAIDs (OR 1.36, 95% CI 1.04 to 1.77) compared to placebo. Selective COX-2 inhibitors (SUCRA = 93.4%) were the safest, followed by anti-NGFs (SUCRA=53.5%) and NSAIDs (SUCRA=50.5%) (Table 3 and Supplementary Table 3). In the subgroup analysis there was no substantial change after excluding the trials that were not commercially funded (Supplementary Table 5). The cluster-rank plots of the primary efficacy and primary safety endpoints showed the selective COX-2 inhibitors to be overall the safest drugs (Supplementary Figure 2).

### Secondary safety endpoint

A total of 33 trials were selected for assessment. Based on incidence rates, the three most common AEs selected as the secondary safety endpoints were nausea, headache, and peripheral sensation abnormality (paresthesia and pruritus).

### Direct pair-wise meta-analysis

There were no significant differences in the incidence rates of headache among the three drug classes from the pair-wise meta-analyses. However, potent opioids and weak opioids had significantly higher risks for nausea and peripheral sensation abnormality. In addition, anti-NGFs had significantly higher risk for peripheral sensation abnormality (Table 3).

### Network meta-analysis

In the headache network, NSAIDs were the safest drugs (OR 0.82, SUCRA 90.3%, 95% CI 0.63 to 1.06). However, there were also no significant differences between the other treatments and placebo. NSAIDs (OR 1.48, 95 % CI 1.04 to 2.13), weak opioids (OR 3.90, 95 % CI 1.82 to 8.36) and potent opioids (OR 6.33, 95 % CI 3.37 to 11.90) had significantly higher risks for nausea. The incidence rate of peripheral sensation abnormality was significantly higher with anti-NGFs (OR 3.64, 95% CI 1.87 to 7.10), weak opioids (OR 5.25, 95% CI 1.95 to 14.15) and potent opioids (OR 5.39, 95% CI 2.41 to 12.06) (Table 3). No subgroup analysis was conducted for secondary safety endpoints because there was an insufficient number of trials.

### Subgroup analysis comparing efficacy of NSAIDs and selective COX-2 inhibitors

A total of 24 trials assessing the efficacy of NSAIDs or **s**elective COX-2 inhibitors were included. All drugs had significantly greater efficacy compared to placebo for both pain relief and function improvement (Figure 2, Supplementary Tables 4, 6) but there were no significant differences between drugs. Etoricoxib produced the highest values for both pain relief (SMD 3.20, 95% CI 1.17 to 5.24) and functional improvement (SMD 3.41, 95% CI 1.88 to 4.93), and based on the cluster-rank plot it was the most efficacious drug (Supplementary Figure 2) However, no significant difference was reported between drugs.

## DISCUSSION

There have been several systematic reviews and meta-analyses comparing the efficacy and safety of anti-NGF drugs with placebo in OA. Schnitzer TJ et al. [46] showed that in knee and hip OA, anti-NGF treatment can provide excellent and superior pain relief and improvement in joint function compared to placebo, and is generally well tolerated with acceptable AEs. Similarly, Chen J et al. [47] confirmed that anti-NGF treatment is superior to placebo in alleviating pain and improving function in knee OA. This current study is the first systematic review and network meta-analysis comparing the efficacy and safety of monoclonal NGF antibodies (anti-NGF drugs) with other drugs commonly used to treat pain associated with OA, including NSAIDs, opioids, and selective COX-2 inhibitors. The Bayesian method used in this study increases the number of studies within each comparison, which in turn increases the robustness and power of the results. Our results showed that in the treatment of OA, 1. monoclonal NGF antibody drugs have the highest overall efficacy, but are not significantly different from selective COX-2 inhibitors, NSAIDs, and opioids; 2. monoclonal NGF antibodies have the highest efficacy for both pain relief and function improvement, while selective COX-2 inhibitors are the safest (the lowest risk of withdrawal related to AEs); 3. among the COX-2 selective inhibitors and NSAIDs, etoricoxib is the most effective for both pain relief and functional improvement based on cluster-rank and SUCRA, but was not significantly different from other selective COX-2 inhibitors and NSAIDs; 4. potent opioids have the lowest efficacy and worst safety (highest risk of withdrawal related to AEs).

A previous network meta-analysis [48] reported that NSAIDs, weak opioids, and potent opioids have similar efficacy for pain relief in OA. However, the study only compared the drugs indirectly via effects versus placebo, and no direct comparisons were made between the drugs, including between the potent opioids and the other drugs. In the current analysis, we included more eligible studies and compared potent opioids directly and indirectly with other drugs. Additionally, we analyzed for functional improvement and safety as well as pain relief, which are all important outcomes in OA treatment. Based on our analyses, potent opioids have low safety and efficacy, and better options are available for treatment of OA.

The new finding from this study is that monoclonal NGF antibodies have the highest efficacy for pain relief and functional improvement, exceeding that of selective COX-2 inhibitors, NSAIDs, and weak opioids (tramadol), all of which are recommended in the 2019^th^ American College of Rheumatology/Arthritis Foundation Guideline. However, monoclonal NGF antibodies have a higher risk of peripheral sensation abnormality (including paresthesia and pruritus), although overall withdrawal rate related to AEs is not significantly different compared to placebo. Our findings are consistent with the results of previous systematic reviews [47, 48] and support monoclonal NGF antibodies as the most effective treatment option for OA. The monoclonal NGF antibodies should be considered the first choice for patients with pain and/or disability related to OA. For patients who experience paresthesia and pruritus, selective COX-2 inhibitors and NSAIDs are preferred treatment options. The choice of a specific COX-2 inhibitor or NSAID can be made based on the mean rank order presented in Supplementary Table 5.

This study has several limitations. Firstly, only randomized, controlled trials (RCTs) were included to avoid uncontrolled confounding factors common in observational studies and other non-RCTs. However, observational studies and other non-RCTs can provide important information on drug efficacy and safety, and omitting these resulted in a small number of studies being included. Secondly, only high-quality studies were included in order to control the quality of the analysis and to minimize the impact of small-sized study effects. However, including small-sized studies can increase generalizability and robustness of the results. Publication bias may be a significant problem, but is difficult to control with a small number of studies. Thirdly, the length of follow-up was not long enough to assess long-term safety outcomes. The median follow-up of studies in this network-analysis of 84 days (IQR 42–84 days) is sufficient to assess AEs that develop relatively quickly but not for long-term safety outcomes such as cardiovascular changes, sensation abnormalities and joint damage. More high-quality RCTs, with long-term follow-up, are needed.

## CONCLUSION

A total of 38 studies comprising 20489 patients were included in this network meta-analysis. The results show that monoclonal NGF antibodies provide significantly greater pain relief and functional improvement in OA compared to selective COX-2 inhibitors, NSAIDs, and opioids. Monoclonal NGF antibodies are not associated with severe short-term AEs. More large scale RCTs are needed to confirm these findings.

## MATERIALS AND METHODS

### Literature search

We searched the PubMed, CNKI, Web of Science, Scopus, Embase and Cochrane Library databases, from the start of January 1999 to end of January 2020. Our search terms were 'osteoarthritis', 'tanezumab', 'fulranumab', 'fasinumab', 'oxycodone', 'hydromorphone', 'oxymorphone', 'tramadol', 'celecoxib', 'etoricoxib', 'rofecoxib', 'ibuprofen', 'naproxen', 'diclofenac', and 'paracetamol/acetaminophen'. We also screened the reference lists of relevant systematic reviews and meta-analyses to identify additional eligible articles. All eligible articles were included irrespective of the language of publication.

### Inclusion/exclusion criteria

The inclusion criteria for this network meta-analysis were: 1. RCTs; 2. studies comparing the target drugs with placebo, or with each other; 3. studies on patients with OA at any joint; 4. studies reporting the following endpoints: pain reduction, functional improvement, and withdrawal related to AEs. The exclusion criteria were: 1. dose-escalation studies of only one drug; 2. target drugs combined with other drugs; 3. studies for postoperative pain; 4. reviews, systematic reviews and meta-analyses, conference abstracts, letters, pharmacokinetic or pharmacodynamic studies, and studies with insufficient data.

For studies with insufficient data, the corresponding authors were contacted to see if the required information could be obtained. If no response was received, a reminder was sent, and if there was still no response the study was excluded. For studies that reported data visually but did not provide numeric values in text or tables, again the corresponding authors were contacted. If no response was received, two authors of the current analysis independently attempted to obtain the data by measurements from the graphs/figures. If that was not possible based on a clear scale and specific reference system then the study was excluded.

### Quality assessment

The Cochrane risk of bias assessment tool was used to perform the methodological quality assessment of the RCTs [49]. The following indices were evaluated and ranked as low risk of bias, unclear risk of bias, or high risk of bias: sequence generation, allocation concealment, blinding, incomplete outcome data, selection outcome reporting, and other sources of bias. These assessments were performed by the authors independently, and all disputes were resolved by discussion among the authors.

### Data extraction

Author, publication year, total sample size, mean age, gender ratio, affected joint, treatments, route of administration, intervention time, follow-up period, and endpoint data were collected and tabulated. To reduce the effect of withdrawal bias, we collected data from the intention-to-treat analysis.

### Outcome measures

The primary efficacy endpoint was pain reduction, and the secondary efficacy endpoint was functional improvement. There were restrictions on the types of questionnaire used in pain evaluation. Functional improvement was evaluated using the function subscale of the Western Ontario and McMaster Universities Arthritis Index (WOMAC). If the WOMAC function score was not measured or reported, the Lequesne Index or other functional measurement scale was used. The change-from-baseline score (mean ± SD) at the last follow-up period was used to evaluate the extent of both pain relief and functional improvement. For studies involving multiple treatment groups with different doses of the same drug, we selected the most effective dose group based on the respective study’s recommendations [50]. We calculated the SMD since results from different scales were used in the same network.

Since patient compliance impacts the effect of treatment in clinical practice, we selected the withdrawal rates related to AEs as the primary safety endpoint. The most commonly related AEs were secondary safety endpoints. We calculated the OR with 95% CI for the safety of target drugs versus placebo or versus each other.

### Statistical analysis

The efficacies of the target drugs were compared to placebo by direct pairwise meta-analyses using Review Manager Software (RevMan. Version 5.3, Copenhagen, the Nordic Cochrane Centre, the Cochrane Collaboration, 2014). Heterogeneity across studies were assessed from the Cochran Q statistic and the I^2^ statistic. When there was significant heterogeneity (P<0.05 or I^2^ >50%), we pooled data and used a random-effects model for analysis. When there was no significant heterogeneity we pooled data and used a fixed-effects model for analysis. The Bayesian network meta-analyses were conducted using Stata/MP (version 14.0, Stata Corp, College Station, Texas, USA) and GeMTC (version 0.14.3). This method increases the number of studies within each comparison and narrows the CI width, resulting in stable results [51–54]. In the Bayesian network meta-analysis, non-informative uniform and normal prior distributions were used. Subsequently, four different sets of starting values were set to fit the model to yield 40000 iterations (10000 per chain) and to obtain the posterior distributions of model parameters [55, 56]. The thinning interval was set at 10 and the burn-ins at 1000, for each chain. Convergence of iterations was assessed using the Gelman-Rubin-Brooks statistic [57]. Global inconsistency tests and local inconsistency tests (Loop-inconsistency tests and Node-split tests) were used to reconfirm the consistency of the network meta-analysis. Two subgroup analyses were conducted. The first was to determine the impact on the results of the network analyses based on whether the trials were commercially funded or not. The second was to determine the most effective drug (vs placebo) for combined pain relief and physical functional improvement (from the selective COX-2 inhibitors celecoxib, etoricoxib, rofecoxib, and the traditional NSAIDs ibuprofen, naproxen, diclofenac, paracetamol/acetaminophen).

All estimate outcomes (SMDs or ORs) with 95% CI were generated from the posterior distribution medians. Differences were considered significant if the 95% CI did not include 0 for SMD or 1 for OR. Differences were considered statistically significant at P<0.05. The minimum clinically important difference of -0.37 SD units was selected. This threshold of 0.37 SD units is based on the median minimum clinically important difference reported in studies in patients with osteoarthritis [58]. An effect size of 0.37 corresponds to a difference of 9 mm on a 100 mm visual analogue scale. The SUCRA was used to rank the efficacy and safety of the different drugs. An intervention with a SUCRA value of 100 is certain to be the best, whereas an intervention with 0 is certain to be the worst [59]. To select the most effective drug based on two or more endpoints, cluster-ranking plots were constructed.

## Supplementary Material

Supplementary Figures

Supplementary Table 1

Supplementary Tables 2-6
