# Supplementary material for: Targeting nerve growth factor, a new option for treatment of osteoarthritis: a network meta-analysis of comparative efficacy and safety with traditional drugs
Source: Aging (Albany NY). 2020 Dec 3;13(1):1051–70. doi: 10.18632/aging.202232 (PMC7835067; doi:10.18632/aging.202232)
Supplement: Supplementary Figures [file aging-13-202232-s001.pdf]

SUPPLEMENTARY FIGURES

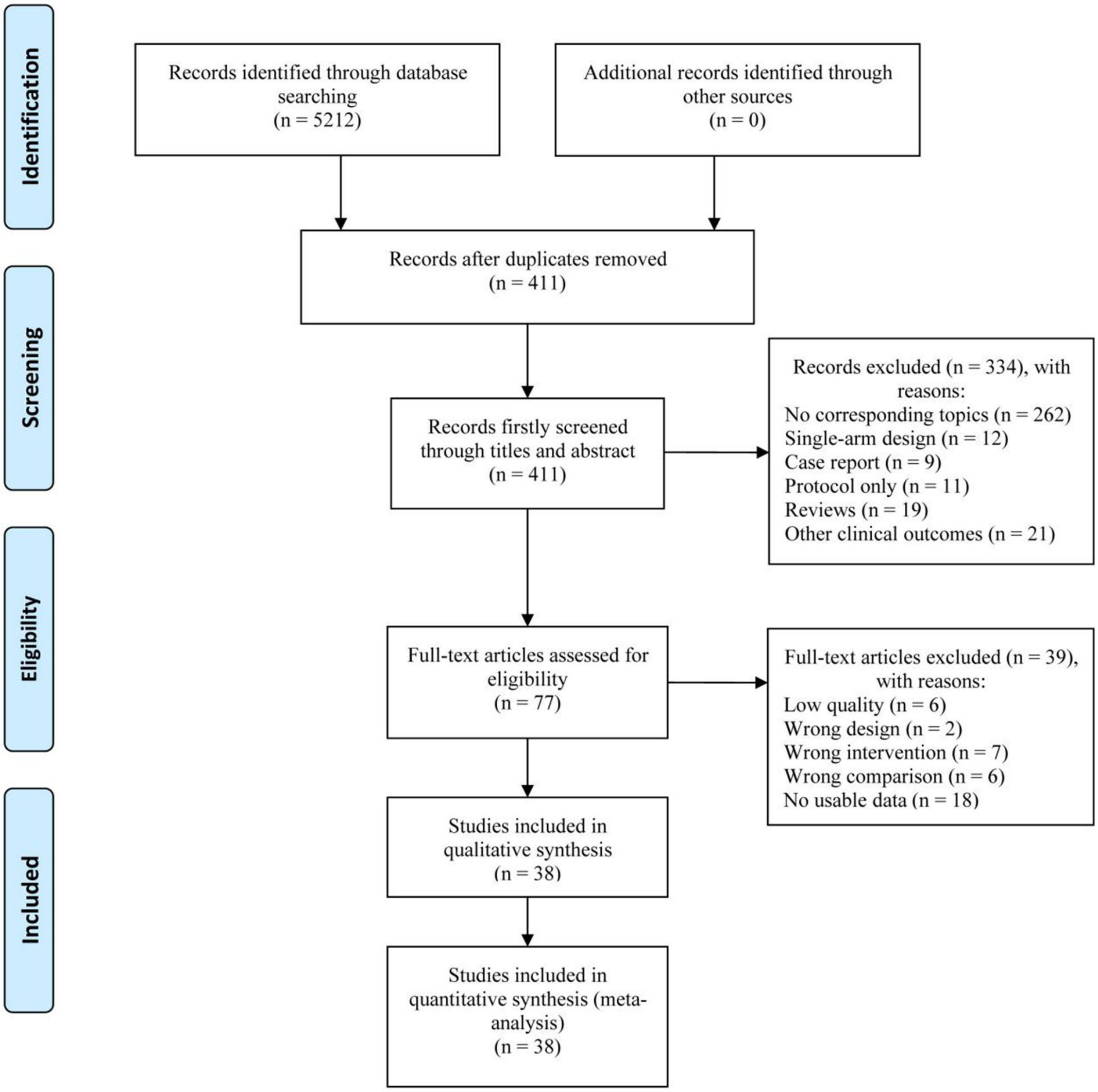

Supplementary Figure 1. PRISMA flow diagram.

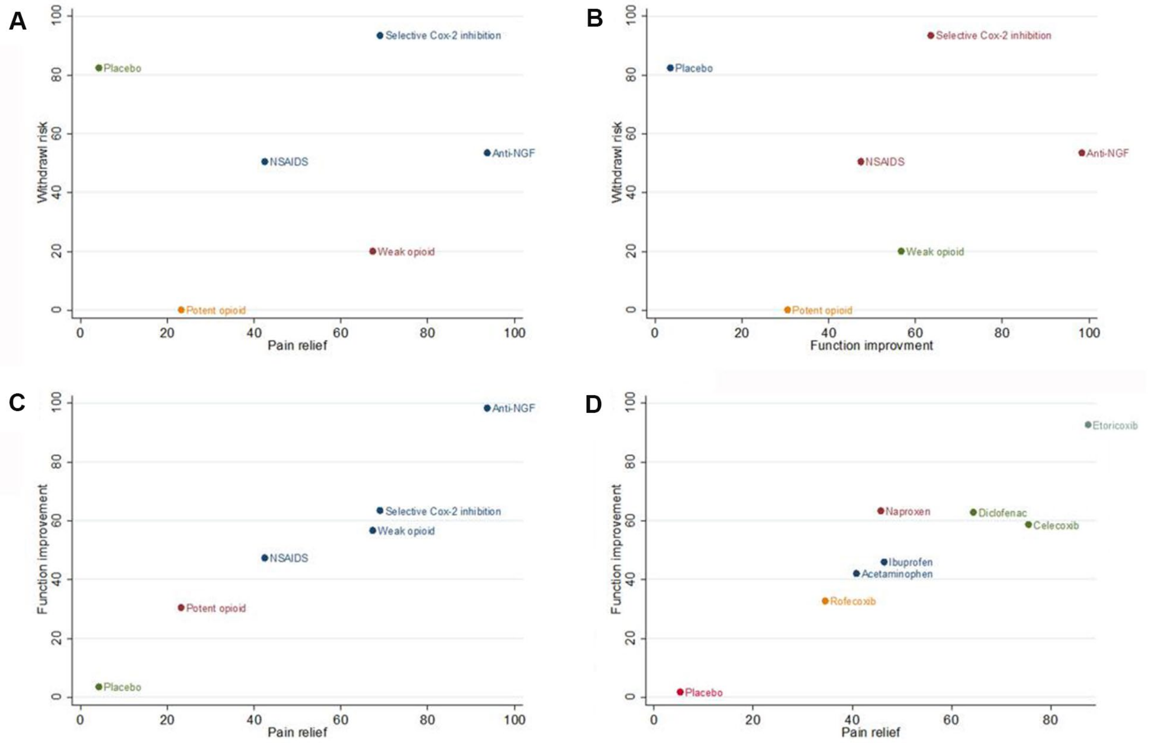

Supplementary Figure 2. Cluster-rank plots. (**A**) The cluster-rank plot of pain relief and withdrawal rate for main network meta-analysis. (**B**) The cluster-rank plot of function improvement and withdrawal rate for main network meta-analysis. (**C**) The cluster-rank plot of pain relief and function improvement for main network meta-analysis. (**D**) The cluster-rank plot of pain relief and function improvement for subgroup analysis comparing different selective COX-2 inhibitor and traditional NSAIDs. (The cluster-rank value is the product of the abscissa and ordinate of each treatment).

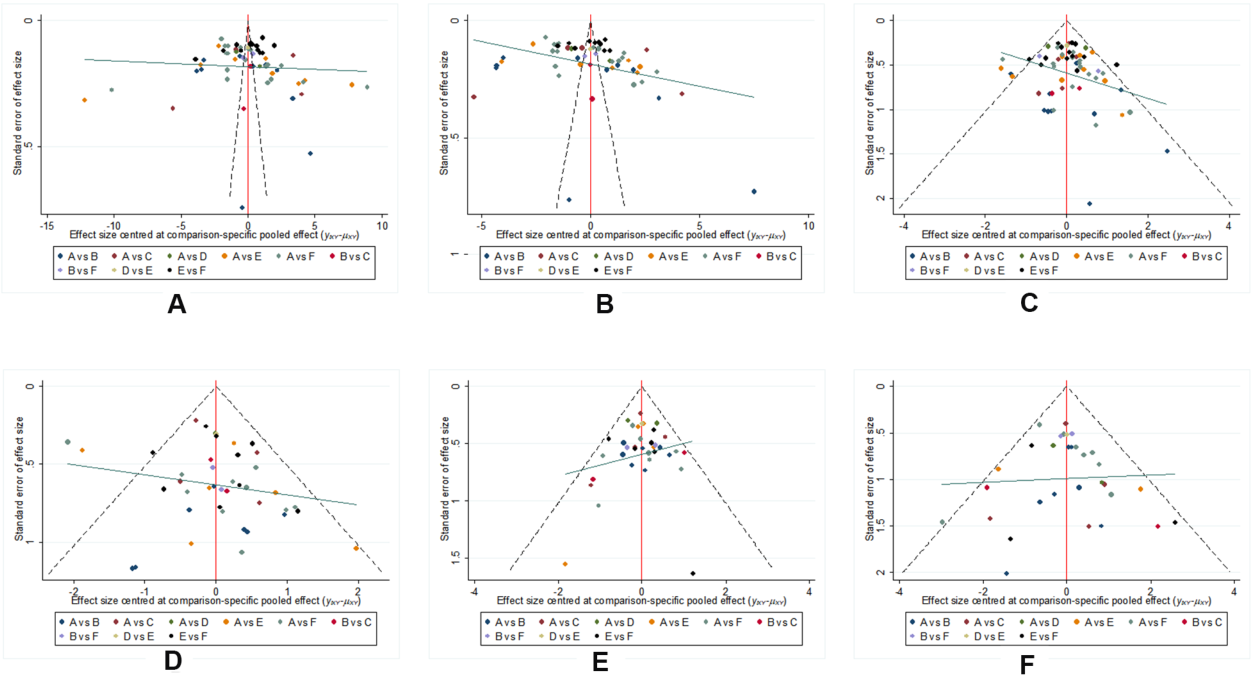

Supplementary Figure 3. Funnel plots. (**A**) The funnel plot of pain relief. (**B**) The funnel plot of function improvement. (**C**) The funnel plot of withdrawal related to AEs. (**D**) The funnel plot of nausea AEs. (**E**) The funnel plot of headache AEs. (**F**) The funnel plot of peripheral sensation abnormality AEs.

```
Testing for inconsistency:
 ( 1)  [_y_B]des_ABC = 0
 ( 2)  [_y_B]des_ABF = 0
 ( 3)  [_y_C]des_AC = 0
 ( 4)  [_y_D]des_ADE = 0
 ( 5)  [_y_E]des_AE = 0
 ( 6)  [_y_F]des_AEF = 0
 ( 7)  [_y_E]des_AEF = 0
 ( 8)  [_y_F]des_AF = 0
 ( 9)  [_y_E]des_EF = 0

           chi2(  9) =   15.06
         Prob > chi2 =    0.0894
```

A

```
Testing for inconsistency:
 ( 1)  [_y_B]des_ABC = 0
 ( 2)  [_y_B]des_ABF = 0
 ( 3)  [_y_C]des_AC = 0
 ( 4)  [_y_D]des_ADE = 0
 ( 5)  [_y_E]des_AE = 0
 ( 6)  [_y_F]des_AEF = 0
 ( 7)  [_y_E]des_AEF = 0
 ( 8)  [_y_F]des_AF = 0
 ( 9)  [_y_E]des_EF = 0

           chi2(  9) =   10.35
         Prob > chi2 =    0.3226
```

B

```
Testing for inconsistency:
 ( 1)  [_y_B]des_ABC = 0
 ( 2)  [_y_B]des_ABF = 0
 ( 3)  [_y_C]des_AC = 0
 ( 4)  [_y_D]des_ADE = 0
 ( 5)  [_y_E]des_AE = 0
 ( 6)  [_y_F]des_AEF = 0
 ( 7)  [_y_E]des_AEF = 0
 ( 8)  [_y_F]des_AF = 0
 ( 9)  [_y_E]des_EF = 0

           chi2(  9) =   13.57
         Prob > chi2 =    0.1385
```

C

```
Testing for inconsistency:
 ( 1)  [_y_B]des_ABC = 0
 ( 2)  [_y_B]des_ABF = 0
 ( 3)  [_y_C]des_AC = 0
 ( 4)  [_y_D]des_ADE = 0
 ( 5)  [_y_F]des_AEF = 0
 ( 6)  [_y_E]des_AEF = 0
 ( 7)  [_y_F]des_AF = 0
 ( 8)  [_y_E]des_EF = 0

           chi2(  8) =    7.48
         Prob > chi2 =    0.4863
```

D

```
Testing for inconsistency:
 ( 1)  [_y_B]des_ABC = 0
 ( 2)  [_y_B]des_ABF = 0
 ( 3)  [_y_C]des_AC = 0
 ( 4)  [_y_D]des_ADE = 0
 ( 5)  [_y_F]des_AEF = 0
 ( 6)  [_y_E]des_AEF = 0
 ( 7)  [_y_F]des_AF = 0
 ( 8)  [_y_E]des_EF = 0

           chi2(  8) =    9.07
         Prob > chi2 =    0.3366
```

E

```
Testing for inconsistency:
 ( 1)  [_y_B]des_ABC = 0
 ( 2)  [_y_B]des_ABF = 0
 ( 3)  [_y_C]des_AC = 0
 ( 4)  [_y_D]des_ADE = 0
 ( 5)  [_y_F]des_AEF = 0
 ( 6)  [_y_E]des_AEF = 0
 ( 7)  [_y_F]des_AF = 0
 ( 8)  [_y_E]des_EF = 0

           chi2(  8) =   11.42
         Prob > chi2 =    0.1789
```

F

Supplementary Figure 4. The details of global inconsistency. The P (prob> chi^2) <0.05 would be considered as significant inconsistency. (**A**) The global inconsistency of pain relief. (**B**) The global inconsistency of function improvement. (**C**) The global inconsistency of withdrawal related to AEs. (**D**) The global inconsistency of nausea AEs. (**E**) The global inconsistency of headache AEs. (**F**) The global inconsistency of peripheral sensation abnormality AEs.

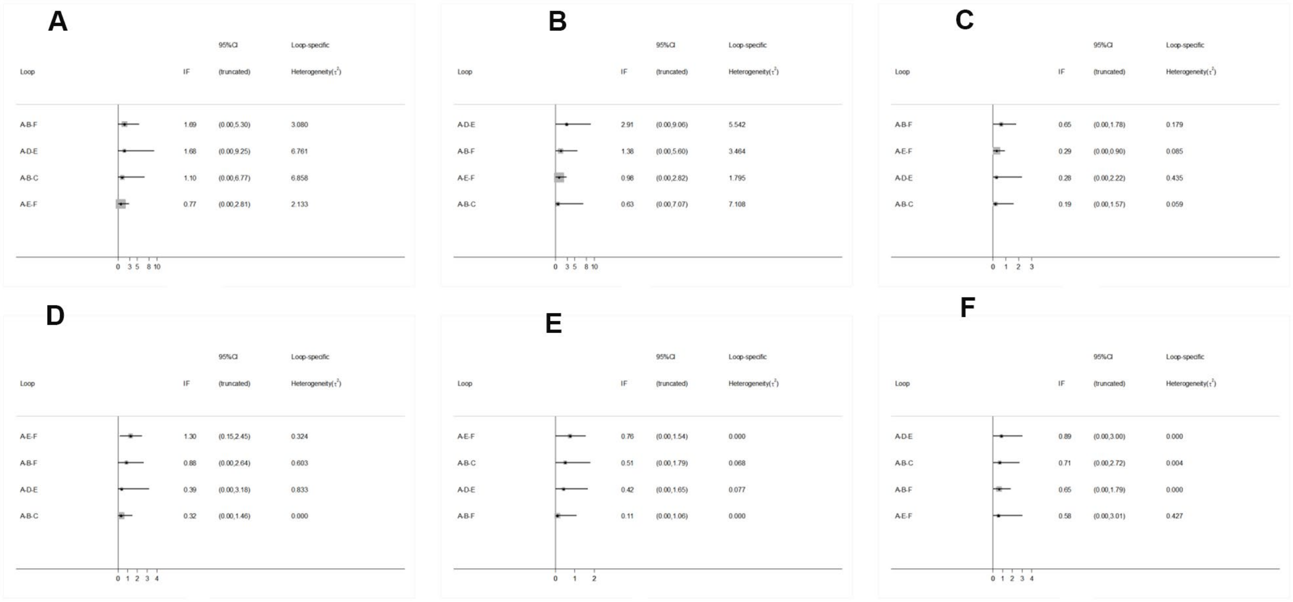

Supplementary Figure 5. The details of local inconsistency. (**A**) The local inconsistency of pain relief. (**B**) The local inconsistency of function improvement. (**C**) The local inconsistency of withdrawal related to AEs. (**D**) The local inconsistency of nausea AEs. (**E**) The local inconsistency of headache AEs. (**F**) The local inconsistency of peripheral sensation abnormality AEs.
