# Supplementary material for: Targeting nerve growth factor, a new option for treatment of osteoarthritis: a network meta-analysis of comparative efficacy and safety with traditional drugs
Source: Aging (Albany NY). 2020 Dec 3;13(1):1051–70. doi: 10.18632/aging.202232 (PMC7835067; doi:10.18632/aging.202232)
Supplement: Supplementary Table 1 [file aging-13-202232-s002.docx]

**Supplementary Appendix Table 1. Baseline characteristics of included studies.**

| **Author** | **No.** | **Year** | **Sample Size** | **Mean age** | **Gender ratio** | **Target joint** | **Route of administration** | **Funded or Sponsored** | **Diagnostic criteria of OA** | BMI, kg/m2 | **Experimental intervention** | **Control intervention I** | **Control intervention II** | **Follow-up period, days** |
| --- | --- | --- | --- | --- | --- | --- | --- | --- | --- | --- | --- | --- | --- | --- |
| Kivitz A et al. | 1 | 2006 | 370 | 61.76 | 146/224 | Hip/Knee | Oral | Yes | Kellgren-Lawrence scale system | NR | Oxymorphone | Placebo | - | 14 |
| Zacher J et al. | 2 | 2003 | 516 | 63.05 | 101/415 | Hip/Knee | Oral | Yes | American College of Rheumatology guideline | NR | Etoricoxib | Diclofenac | - | 42 |
| Spierings EL et al. | 3 | 2013 | 610 | 57.41 | 229/381 | Hip/Knee | Itravenous | Yes | Kellgren-Lawrence scale system | NR | Placebo | Tanezumab | Oxycodone | 112 |
| Puopolo A et al. | 4 | 2007 | 548 | 62.56 | 133/415 | Hip/Knee | Oral | No | American Rheumatism Association guideline | NR | Placebo | Etoricoxib | Ibuprofen | 84 |
| Vojtaššák J et al. | 5 | 2011 | 287 | 65.52 | 80/207 | Hip/Knee | Oral | No | American College of Rheumatology guideline | 30.57(5.35) | Hydromorphone | Placebo | - | 112 |
| Prior MJ et al. | 6 | 2014 | 542 | 61.7 | 139/403 | Hip/Knee | Oral | No | Kellgren-Lawrence scale system | NR | Acetaminophen | Placebo | - | 84 |
| Emery P et al. | 7 | 2008 | 248 | 64.02 | 134/114 | Hip | Oral | Yes | American College of Rheumatology guideline | NR | Celecoxib | Diclofenac | - | 84 |
| McKenna F et al. | 8 | 2001 | 600 | 61.67 | 208/392 | Knee | Oral | Yes | American College of Rheumatology guideline | NR | Placebo | Celecoxib | Diclofenac | 42 |
| Altman RD et al. | 9 | 2015 | 385 | 64.13 | 89/296 | Hand | Topical | No | American College of Rheumatology guideline | 28.29(6.41) | Diclofenac | Placebo | - | 56 |
| Bookman AA et al. | 10 | 2004 | 248 | 61.8 | 91/157 | Knee | Topical | No | NR | NR | Diclofenac | Placebo | - | 28 |
| Schnitzer TJ et al. | 11 | 2019 | 696 | 60.83 | 243/453 | Hip/Knee | Itravenous | Yes | American College of Rheumatology guideline | NR | Tanezumab | Placebo | - | 112 |
| Mayorga AJ et al. | 12 | 2016 | 196 | NR | 86/110 | Knee | Itravenous | Yes | American College of Rheumatology guideline | 31.49(4.92) | Placebo | Fulranumab | Oxycodone | 84 |
| Ekman EF et al. | 13 | 2014 | 828 | 61.12 | 329/499 | Knee | Itravenous | Yes | American College of Rheumatology guideline | 30.90(4.69) | Placebo | Tanezumab 5mg,10mg | Naproxen | 56 |
| Ekman EF et al. | 14 | 2014 | 840 | 59.85 | 304/534 | Hip/Knee | Itravenous | Yes | American College of Rheumatology guideline | 30.33(4.82) | Placebo | Tanezumab 5mg,10mg | Naproxen | 56 |
| Gibofsky A et al. | 15 | 2014 | 305 | 61.63 | 102/203 | Hip/Knee | Oral | Yes | Kellgren-Lawrence scale system | 31.49(5.19) | Diclofenac | Placebo | - | 84 |
| Essex MN et al. | 16 | 2016 | 367 | 64.77 | 120/247 | Knee | Oral | Yes | American Rheumatism Association guideline | NR | Celecoxib | Naproxen | Placebo | 42 |
| Reed K et al. | 17 | 2018 | 676 | 60.77 | 251/425 | Hip/Knee | Oral | Yes | American College of Rheumatology guideline | 30.3(4.77) | Acetaminophen | Placebo | - | 84 |
| Essex MN et al. | 18 | 2012 | 589 | 60.38 | 199/390 | Knee | Oral | Yes | American College of Rheumatology guideline | NR | Celecoxib | Naproxen | - | 180 |
| Leung AT et al. | 19 | 2002 | 501 | 63.16 | 109/392 | Hip/Knee | Oral | Yes | American Rheumatism Association guideline | NR | Placebo | Etoricoxib | Naproxen | 84 |
| Gordo AC et al. | 20 | 2016 | 388 | 62.95 | 106/282 | Knee | Oral | Yes | American College of Rheumatology guideline | NR | Celecoxib | Ibuprofen | Placebo | 42 |
| Niethard FU et al. | 21 | 2005 | 237 | 66 | 87/150 | Knee | Topical | No | NR | NR | Diclofenac（Gel） | Placebo | - | 21 |
| Tiseo PJ et al. | 22 | 2014 | 215 | 59.3 | 67/148 | Knee | Itravenous | Yes | American College of Rheumatology guideline | 31.30(5.50) | Placebo | Fasinumab | - | 168 |
| Yoo MC et al. | 23 | 2014 | 239 | 63 | 25/214 | Knee | Oral | Yes | American Rheumatism Association guideline | 25.20(2.85) | Etoricoxib | Celecoxib | - | 84 |
| DeLemos BP et al. | 24 | 2011 | 1001 | 60.02 | 369/632 | Hip/Knee | Oral | Yes | American College of Rheumatology guideline | NR | Tramadol | Celecoxib | Placebo | 84 |
| Reginster JY et al. | 25 | 2006 | 997 | 62.77 | 279/718 | Hip/Knee | Oral | No | American Rheumatism Association guideline | NR | Placebo | Etoricoxib | Naproxen | 84 |
| Reginster JY et al. | 26 | 2006 | 463 | 65.7 | 116/347 | Hip/Knee | Oral | No | American Rheumatism Association guideline | NR | Etoricoxib | Naproxen | - | 280 |
| Gana TJ et al. | 27 | 2006 | 1011 | 58.15 | 380/631 | Hip/Knee | Oral | Yes | American College of Rheumatology guideline | NR | Placebo | Tramadol | - | 84 |
| Svensson O et al. | 28 | 2006 | 156 | 58.66 | 49/107 | Hip | Oral | Yes | American College of Rheumatology guideline | 31.00(5.33) | Naproxen | Placebo | - | 42 |
| Svensson O et al. | 29 | 2006 | 355 | 60.11 | 84/271 | Knee | Oral | Yes | American College of Rheumatology guideline | 26.00(4.54) | Naproxen | Placebo | - | 42 |
| Nagashima H et al. | 30 | 2011 | 83 | 59.03 | 25/57 | Knee | Itravenous | Yes | American College of Rheumatology guideline | NR | Tanezumab | Placebo | - | 56 |
| Dakin P et al. | 31 | 2019 | 421 | 60.6 | 149/272 | Hip/Knee | Itravenous | Yes | American College of Rheumatology guideline | 31.12(4.9) | Placebo | Fasinumab | - | 112 |
| Brown MT et al. | 32 | 2013 | 621 | 62.32 | 237/384 | Hip | Itravenous | Yes | Kellgren-Lawrence scale system | NR | Placebo | Tanezumab | - | 224 |
| Lane NE et al. | 33 | 2010 | 444 | 58.7 | 182/262 | Knee | Itravenous | Yes | American College of Rheumatology guideline | NR | Placebo | Tanezumab | - | 112 |
| Rauck R et al. | 34 | 2012 | 981 | 59.7 | 354/627 | Hip/Knee | Oral | Yes | Kellgren-Lawrence scale system | 34.20(7.8) | Placebo | Hydromorphone | - | 84 |
| Miceli-Richard C et al. | 35 | 2015 | 779 | 70 | 196/583 | Knee | Oral | No | Kellgren-Lawrence scale system | 29.00(5.00) | Acetaminophen | Placebo | - | 42 |
| Varadi G et al. | 36 | 2013 | 75 | 61.28 | 27/48 | Knee | Topical | Yes | Kellgren-Lawrence scale system | 27.97(3.13) | Ibuprofen | Placebo | - | 14 |
| Altman RD et al. | 37 | 2006 | 483 | 62.2 | 160/323 | Hip/Knee | Oral | Yes | American College of Rheumatology guideline | 32.70(8.31) | Acetaminophen | Placebo | - | 84 |
| Schnitzer TJ et al. | 38 | 2009 | 376 | 59.77 | 141/235 | Knee | Oral | Yes | American College of Rheumatology guideline | 33.02(8.02) | Acetaminophen | Rofecoxib | - | 28 |
| Day R et al. | 39 | 2000 | 809 | 63.86 | 162/647 | Hip/Knee | Oral | Yes | American Rheumatism Association guideline | NR | Placebo | Rofecoxib 12.5mg Qd,25mg Qd | Ibuprofen | 42 |
| Ehrich EW et al. | 40 | 1999 | 219 | 63.5 | 63/156 | Knee | Oral | No | American Rheumatism Association guideline | NR | Placebo | Rofecoxib | - | 42 |
| Cannon GW et al. | 41 | 2000 | 784 | 63.6 | 255/529 | Hip/Knee | Oral | No | American College of Rheumatology guideline | NR | Rofecoxib | Diclofenac | - | 365 |
