# Supplementary material for: Targeting nerve growth factor, a new option for treatment of osteoarthritis: a network meta-analysis of comparative efficacy and safety with traditional drugs
Source: Aging (Albany NY). 2020 Dec 3;13(1):1051–70. doi: 10.18632/aging.202232 (PMC7835067; doi:10.18632/aging.202232)
Supplement: Supplementary Tables 2-6 [file aging-13-202232-s003.pdf]

SUPPLEMENTARY TABLES

Supplementary Table 2. Methodological quality and risk of bias evaluation.

Article	1.Sequence generation	2.Allocation concealment	3.Blinding	4.Incomplete outcome data	5.Selective outcome reporting	6.Other source of bias
Kivitz A et al.	Low risk	Low risk	Low risk	Low risk	Low risk	High risk
Zacher J et al.	Low risk	Low risk	Low risk	Low risk	Low risk	Low risk
Spierings EL et al.	High risk	Low risk	Low risk	Low risk	Low risk	Unclear risk
Puopolo A et al.	Low risk	Low risk	Low risk	Low risk	Low risk	Unclear risk
Vojtaššák J et al.	Low risk	Low risk	Low risk	Low risk	Low risk	Unclear risk
Prior MJ et al.	Low risk	Low risk	Low risk	Low risk	Low risk	Unclear risk
Emery P et al.	Low risk	Low risk	Low risk	Low risk	Low risk	Unclear risk
McKenna F et al.	Low risk	Low risk	Low risk	Low risk	Low risk	Unclear risk
Altman RD et al.	Unclear risk	Low risk	Low risk	Low risk	Low risk	Unclear risk
Bookman AA et al.	Unclear risk	Low risk	Low risk	Low risk	Low risk	Low risk
Schnitzer TJ et al.	Low risk	Low risk	Low risk	Low risk	Low risk	Unclear risk
Mayorga AJ et al.	Low risk	Low risk	Low risk	Low risk	Low risk	Unclear risk
Ekman EF et al.	Low risk	Low risk	Low risk	Low risk	Low risk	Unclear risk
Ekman EF et al.	Low risk	Low risk	Low risk	Low risk	Low risk	Unclear risk
Gibofsky A et al.	Unclear risk	Low risk	Low risk	Low risk	Low risk	Unclear risk
Essex MN et al.	Unclear risk	Low risk	Low risk	Low risk	Low risk	Unclear risk
Reed K et al.	Low risk	Low risk	Low risk	Low risk	Low risk	Unclear risk
Essex MN et al.	Low risk	Low risk	Low risk	Low risk	Low risk	Unclear risk
Leung AT et al.	Low risk	Low risk	Low risk	Low risk	Low risk	Unclear risk
Gordo AC et al.	Low risk	Low risk	Low risk	Low risk	Low risk	Unclear risk
Niethard FU et al.	Low risk	Low risk	Low risk	Low risk	Low risk	High risk
Tiseo PJ et al.	Low risk	Low risk	Low risk	Low risk	Low risk	Unclear risk
Yoo MC et al.	Low risk	Unclear risk	Low risk	Low risk	Low risk	Unclear risk
DeLemos BP et al.	Unclear risk	Low risk	Low risk	Low risk	Low risk	Low risk
Reginster JY et al.	Low risk	Low risk	Low risk	Low risk	Low risk	Low risk
Reginster JY et al.	Low risk	Low risk	Low risk	Low risk	Low risk	Low risk
Gana TJ et al.	Low risk	Low risk	Low risk	Low risk	Low risk	Low risk
Svensson O et al.	Unclear risk	Unclear risk	Low risk	Low risk	Low risk	Low risk
Svensson O et al.	Unclear risk	Unclear risk	Low risk	Low risk	Low risk	Low risk
Nagashima H et al.	High risk	Low risk	Low risk	Low risk	Low risk	Unclear risk
Dakin P et al.	Low risk	Low risk	Low risk	Low risk	Low risk	Unclear risk
Brown MT et al.	Unclear risk	Low risk	Low risk	Low risk	Low risk	Unclear risk
Lane NE et al.	Low risk	Low risk	Low risk	Low risk	Low risk	Unclear risk
Rauck R et al.	Unclear risk	Low risk	Low risk	Low risk	Low risk	Unclear risk
Miceli-Richard C et al.	Unclear risk	Unclear risk	Low risk	Low risk	Low risk	Unclear risk
Varadi G et al.	Unclear risk	Unclear risk	Low risk	Low risk	Low risk	Unclear risk

Altman RD et al.	Low risk	Low risk	Low risk	Low risk	Low risk	Unclear risk
Schnitzer TJ et al.	Unclear risk	Low risk	Low risk	Low risk	Low risk	Unclear risk
Day R et al.	Low risk	Low risk	Low risk	Low risk	Low risk	Low risk
Ehrich EW et al.	Low risk	Low risk	Low risk	Low risk	Low risk	Unclear risk
Cannon GW et al.	Low risk	Low risk	Low risk	Low risk	Low risk	Unclear risk

Supplementary Table 3. The details of SURCA and mean rank of main network meta-analysis.

Treatment	SMD (95%CI) for pain relief	SURCA for pain relief, %	Mean rank for pain relief	SMD (95%CI) for function improvement	SURCA for function improvement, %	Mean rank for function improvement
Placebo	Reference	4.2	5.8	Reference	3.5	5.8
Anti-NGF	4.25 (2.87,5.63)	93.7	1.3	4.90 (3.46,6.33)	98.3	1.1
Potent opioids	0.90 (-1.04,2.84)	23.2	4.8	1.20 (-0.81,3.21)	30.5	4.5
Weak opioids	3.03 (0.04,6.02)	67.3	2.6	2.42 (-0.68,5.52)	56.7	3.2
Selective cox-2 inhibition	2.92 (1.58,4.26)	69	2.5	2.56 (1.17,3.95)	63.5	2.8
NSAIDs	1.92 (0.92,2.93)	42.5	3.9	2.03 (0.97,3.09)	47.4	3.6

Supplementary Table 4. The details of SURCA and mean rank of subgroup analysis comparing different selective COX-2 inhibitor and traditional NSAIDs.

Treatment	SMD (95%CI) for pain relief	SURCA for pain relief, %	Mean rank for pain relief	SMD (95%CI) for function improvement	SURCA for function improvement, %	Mean rank for function improvement
Placebo	Reference	5.3	7.6	Reference	1.7	7.9
Acetaminophen	1.42 (-0.31,3.15)	40.8	5.1	1.71 (0.23,3.20)	42	5.1
Celecoxib	2.62 (1.00,4.23)	75.5	2.7	2.26 (0.89,3.63)	58.7	3.9
Diclofenac	2.21 (0.80,3.62)	64.4	3.5	2.35 (1.15,3.55)	62.9	3.6
Etoricoxib	3.20 (1.17,5.24)	87.5	1.9	3.41 (1.88,4.93)	92.7	1.5
Ibuprofen	1.59 (-0.90,4.08)	46.4	4.8	1.87 (0.14,3.60)	46	4.8
Naproxen	1.65 (0.01,3.28)	45.7	4.8	2.38 (0.86,3.89)	63.4	3.6
Rofecoxib	1.16 (-1.13,3.44)	34.5	5.6	1.38 (-0.58,3.35)	32.7	5.7

Supplementary Table 5. The details of results for subgroup analysis of commercial funding.

Treatment	Network-meta analysis			Subgroup analysis		
	SMD (95%CI) for pain relief	SMD (95%CI) for function improvement	OR (95%CI) for withdrawal due to AEs	SMD (95%CI) for pain relief	SMD (95%CI) for function improvement	OR (95%CI) for withdrawal due to AEs
Anti-NGF	4.25 (2.87,5.63)	4.90 (3.46,6.33)	1.36 (0.82,2.27)	4.30 (2.85,5.74)	4.96 (3.42,6.50)	0.36 (-0.22,0.94)
Potent opioids	0.90 (-1.04,2.84)	1.20 (-0.81,3.21)	8.63 (5.42,13.77)	1.13 (-1.10,3.36)	1.47 (-0.90,3.84)	2.19 (1.54,2.84)
Weak opioids	3.03 (0.04,6.02)	2.42 (-0.68,5.52)	3.27 (1.89,5.66)	3.07 (-0.05,6.20)	2.47 (-0.85,5.79)	1.49 (1.04,1.93)
Selective cox-2 inhibition	2.92 (1.58,4.26)	2.56 (1.17,3.95)	0.89 (0.64,1.24)	3.07 (1.40,4.74)	2.75 (0.98,4.53)	-0.10 (-0.58,0.37)
NSAIDs	1.92 (0.92,2.93)	2.03 (0.97,3.09)	1.36 (1.04,1.77)	2.08 (0.80,3.36)	2.22 (0.83,3.61)	0.39 (-0.01,0.80)

Supplementary Table 6. Detailed results of network meta-analysis for pain (Red) and function (Blue) (Data are standardised mean difference, from the top left to the bottom right, higher comparator vs lower comparator, and their related 95% CI).

Etoricoxib	1.15 (-0.56,2.86)	1.06 (-0.65,2.76)	1.53 (-0.52,3.59)	1.03 (-0.68,2.74)	1.69 (-0.42,3.81)	2.02 (-0.40,4.45)	3.41 (1.88,4.93)
0.59 (-1.54,2.72)	Celecoxib	-0.09 (-1.64,1.45)	0.38 (-1.60,2.37)	-0.12 (-1.87,1.63)	0.55 (-1.46,2.55)	0.88 (-1.45,3.20)	2.26 (0.89,3.63)
0.99 (-1.16,3.14)	0.40 (-1.39,2.20)	Diclofenac	-0.48 (-2.50,1.55)	-0.03 (-1.84,1.79)	-0.64 (-2.49,1.22)	-0.97 (-3.03,1.10)	-2.35 (-3.55,-1.15)
1.61 (-1.50,4.73)	1.03 (-1.68,3.73)	0.62 (-2.17,3.42)	Ibuprofen	-0.50 (-2.69,1.68)	-0.16 (-2.44,2.11)	-0.49 (-3.09,2.10)	-1.87 (-3.60,-0.14)
1.56 (-0.48,3.60)	0.97 (-0.87,2.81)	0.57 (-1.41,2.55)	-0.05 (-2.94,2.83)	Naproxen	-0.66 (-2.78,1.45)	-0.99 (-3.44,1.45)	-2.38 (-3.89,-0.86)
1.79 (-0.87,4.44)	1.20 (-1.15,3.54)	0.80 (-1.37,2.96)	0.17 (-2.85,3.20)	0.23 (-2.14,2.59)	Acetaminophen	-0.33 (-2.46,1.80)	-1.71 (-3.20,-0.23)
2.05 (-0.92,5.02)	1.46 (-1.25,4.17)	1.06 (-1.35,3.46)	0.43 (-2.92,3.79)	0.49 (-2.27,3.25)	0.26 (-2.21,2.74)	Rofecoxib	-1.38 (-3.35,0.58)
3.20 (1.17,5.24)	2.62 (1.00,4.23)	2.21 (0.80,3.62)	1.59 (-0.90,4.08)	1.65 (0.01,3.28)	1.42 (-0.31,3.15)	1.16 (-1.13,3.44)	Placebo
